# Advanced porous materials and emerging technologies for radionuclides removal from Fukushima radioactive water

**DOI:** 10.1016/j.eehl.2023.09.001

**Published:** 2023-09-09

**Authors:** Xiaolu Liu, Muliang Xiao, Yang Li, Zhongshan Chen, Hui Yang, Xiangke Wang

**Affiliations:** MOE Key Laboratory of Resources and Environmental System Optimization, College of Environmental Science and Engineering, North China Electric Power University, Beijing 102206, China

**Keywords:** Radioactive water, Radionuclides removal, Advanced porous materials, Emerging technologies

## Abstract

Japan recently announced the plan to discharge over 1.2 million tons of radioactive water into the Pacific Ocean, which contained hazardous radionuclides such as ^60^Co, ^90^Sr, ^125^Sb, ^129^I, ^3^H, ^137^Cs, and ^99^TcO_4_^−^, etc. The contaminated water will pose an enormous threat to global ecosystems and human health. Developing materials and technologies for efficient radionuclide removal is highly desirable and arduous because of the extreme conditions, including super acidity or alkalinity, high ionic strength, and strong ionizing radiation. Recently, advanced porous material, such as porous POPs, MOFs, COFs, PAFs, etc., has shown promise of improved separation of radionuclides due to their intrinsic structural advantages. Furthermore, emerging technologies applied to radionuclide removal have also been summarized. In order to better deal with radionuclide contamination, higher requirements for the design of nanomaterials and technologies applied to practical radionuclide removal are proposed. Finally, we call for comprehensive implementation of strategies and strengthened cooperation to mitigate the harm caused by radioactive contamination to oceans, atmosphere, soil, and human health.

## Highlights

• Japan recently announced the plan to discharge over 1.2 million tons of radioactive water into the Pacific Ocean.
• Radionuclide-contaminated water will pose an enormous threat to the global ecosystems and human health.
• Advanced porous material and emerging technologies applied to radionuclides removal have been summarized.
• Higher requirements for the design of nanomaterials and technologies applied to the practical radionuclide removal are proposed.

## Graphical abstract

**Figure:**
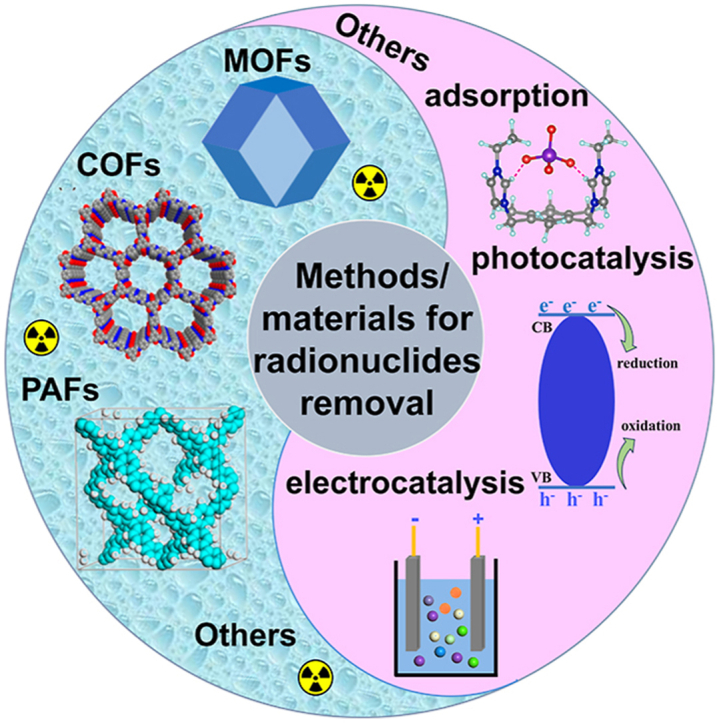

## 1 Introduction

Plentiful radionuclides were released into the surrounding environment after the Fukushima nuclear accident (FNA) in Japan in 2011, which posed an enormous threat to global ecosystems and public health [[1], [2], [3]]. Japan has formulated a medium- and long-term decommissioning plan for the FNA power plant (30–40 years), including radioactive water treatment, nuclear fuel removal, and other waste treatment [4]. The operators of Tokyo Electric Power Company (TEPCO) have been pumping water to cool the nuclear reactors in FNA for the past 10 years, leading to an abundance of radioactive water stored in many large tanks [5]. Up to December 2020, over 1.2 million tons of radioactive water have been produced and stored in large tanks, and about 1,000 tanks will run out of space [6]. In April 2021, the Japanese government announced that they would dump radioactive water from the FNA into the Pacific Ocean two years later. The decision immediately triggered widespread discussion and concern all over the world and was violently opposed by many countries, international organizations, local residents, and fishermen.

Radioactive water has been treated by an advanced liquid processing system (ALPS) before being discharged, which still contained radionuclides with comparatively long half-lives, such as ^3^H (half-life of 12.3 years), ^14^C (half-life of 5,730 years), ^60^Co (half-life of 5.26 years), ^90^Sr (half-life of 28.79 years), ^125^Sb (half-life of 2.71 years), ^129^I (half-life of 1.57 × 10^7^ years), ^137^Cs (half-life of 30.2 years), etc. [7]. Table 1 shows the concentration of 13 radionuclides in Fukushima’s radioactive water before and after ALPS treatment [7,8]. The concentration of radionuclides with a half-life of less than 2 years (^54^Mn, ^89^Sr, ^106^Ru, ^106^Rh, ^134^Cs) can be reduced by more than 96% after 10 years of storage. However, radionuclides with long half-lives need to be removed by chemical/physical separation, and special attention should be paid to the removal effect of radionuclides and the hazards after discharge into the ocean. Notably, the concentration of ^3^H remained unchanged before and after ALPS treatment, which means that all ^3^H in the radioactive water will be discharged into the sea. ^3^H has no external radiation harm to humans, but can easily enter the human body through the food chain, resulting in internal radiation harm and threatening human health. Treatment techniques for wastewater with high ^3^H concentration include chemical exchange, low-temperature distillation, electrochemistry, solvent extraction, etc. Of particular concern is the immaturity of technology, low frequency of filter replacement, chemical composition difference of radioactive water, and operational troubles in the early stage of Fukushima radioactive water treatment, resulting in the concentration of radionuclides such as ^3^H, ^129^I, ^90^Sr, etc. higher than the emission concentration limit in the law of Japan [7,8]. In addition, the concentration level of other highly toxic radionuclides, such as ^239^Pu, ^240^Pu, and ^241^Am, etc., in Fukushima radioactive water was not disclosed by TEPCO. These radionuclides will contaminate the oceans, atmosphere, and soil, and threaten human health. Therefore, developing advanced nanomaterials and technologies for efficient removal of radionuclides to avoid potential harm is urgent and desired.

**Table 1:** Radionuclide concentrations in the ALPS-treated Fukushima radioactive wastewater in comparison with concentration limits required by Japan law, guidance levels for radionuclides in drinking water provided by the World Health Organization (WHO) [7,8].

Radionuclide	Half-life period (year)	ALPS (Bq/L)		Allowable emission concentration limit in law of Japan (Bq/L)	Concentrations in drinking water in WHO (Bq/L)
		Concentration before ALPS	Concentration after ALPS		
^3^H	12.3	10^5^–10^7^	10^5^–10^7^	6 × 10^4^	10^4^
^14^C	5,730	10–100	10–100	2 × 10^3^	100
^54^Mn	0.85	10–10^4^	<∼0.1	10^3^	100
^60^Co	5.27	10–10^5^	<∼0.1–100	200	100
^89^Sr	0.14	<10^3^–10^6^	<0.1	300	100
^90^Sr	28.8	10^4^–10^8^	<∼0.1–10^4^	30	10
^99^Tc	2.14 × 10^5^	1–100	<1	10^3^	100
^106^Ru	1.0	10^2^–10^5^	<1–10^3^	100	10
^106^Rh	29.8 s	10^4^–10^5^	10–100	3 × 10^5^	10^3^
^125^Sb	2.71	10^2^–10^6^	<1–10^3^	800	100
^129^I	1.57 × 10^7^	10^2^–10^3^	<∼0.1–10^3^	9	1
^134^Cs	2.06	10^2^–10^5^	<∼0.1–100	60	10
^137^Cs	30.2	10^2^–10^5^	<∼0.1–100	90	10

## 2 Advanced porous material for radionuclide removal

**Fig. 1:**
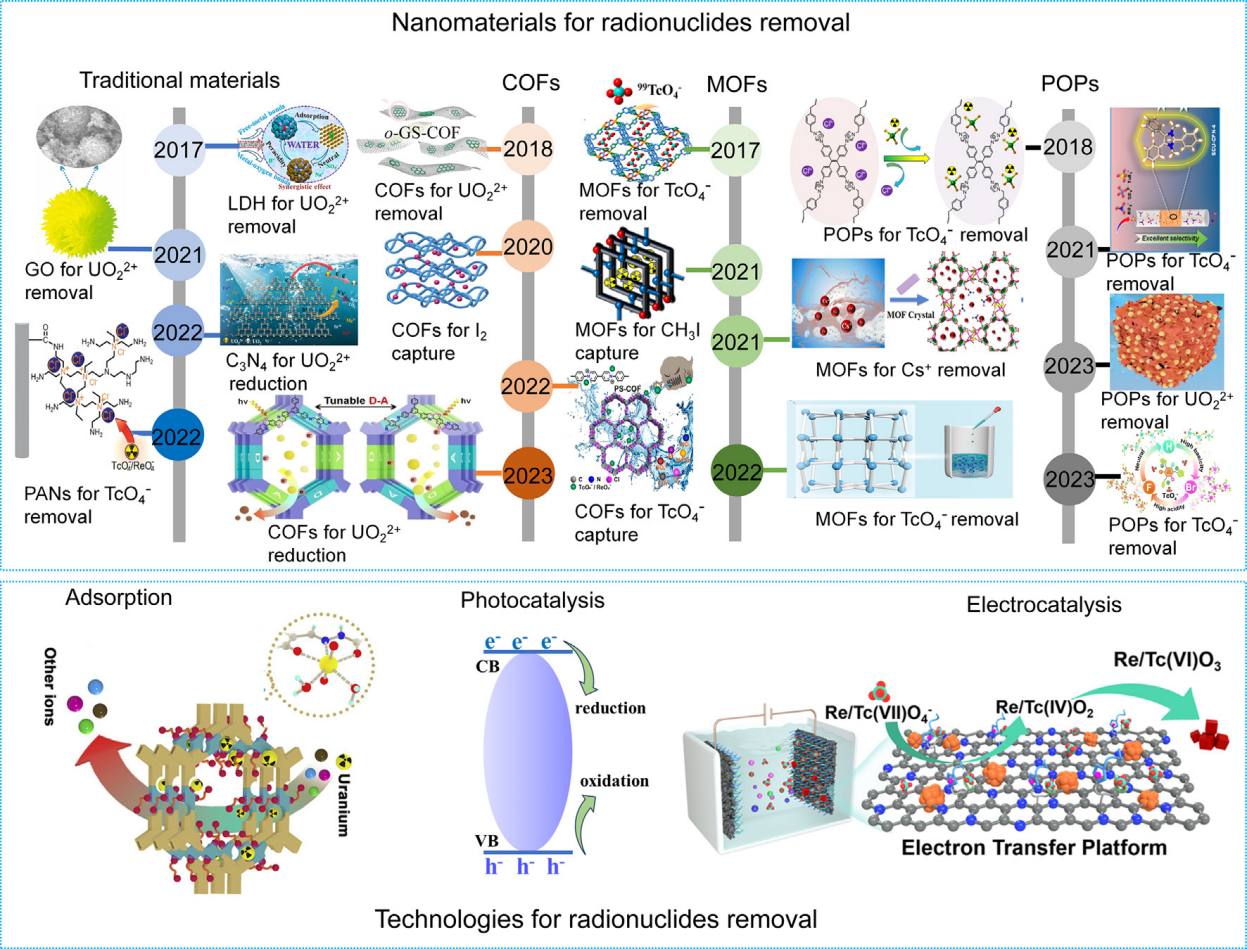
Advanced porous material and emerging technologies for radionuclide removal. The structure of materials was cited from refs. [10,11,15,22,27,[34], [35], [36], [37], [38], [39], [40], [41], [42], [43], [44], [45], [46]].

Traditional materials, such as clay minerals, activated carbon (AC), carbon nanotubes (CNTs), resin, and layered double hydroxides (LDHs), have been extensively used to remove radionuclides (UO_2_^2+^, TcO_4_^−^, I_2_, Cs^+^, Sr^2+^, etc.), but showed limitations such as low adsorption kinetics, poor selectivity, or limited adsorption capacity [9]. Advanced porous materials, such as porous organic polymers (POPs) [10,11], metal–organic frameworks (MOFs) [12], covalent organic frameworks (COFs) [13], porous aromatic frameworks (PAFs) [14], with specialties of high specific surface area, abundant pore structure, high stability, and structural designability, are promising candidates for various radionuclides removal (Fig. 1). Based on previous research, our team recently reported a study that achieved efficient removal of ^99^TcO_4_^−^/ReO_4_^−^ under different conditions by modulating halogen anions in POPs [15]. iCOP-1 contained imidazolium-N^+^ nanotraps demonstrated fast kinetics (up to adsorption equilibrium with 60 s), large adsorption capacity (1,434.1 ± 24.6 mg/g), and excellent selectivity for the removal of ^99^TcO_4_^−^ and ReO_4_^−^. By introducing F or Br near the imidazole-N^+^ nanotraps, efficient removal of ReO_4_^−^ under strong acid or super alkaline conditions can be achieved, respectively. The introduction of F and Br improved the hydrophobicity and strong steric effects of iCOP-1, respectively, thereby achieving efficient removal of ReO_4_^−^/^99^TcO_4_^−^ under different conditions. ^90^Sr, as a β-emitting radioisotope, showed high toxicity and great harm to humans and the environment. Wang et al. successfully prepared a stable uranyl organic framework material, which exhibited significant umbellate distortions in the uranyl equatorial planes, enabling the effective removal of cesium from aqueous solutions [16]. Zhang et al. designed and synthesized the coordination polymer and crystalline zirconium phosphonate, realizing the high selectivity and high efficiency of Sr^2+^ removal under acidic and alkaline conditions, respectively [17,18].

In addition to nanoporous materials designed for the removal of radionuclides in solution, the design and development of materials for radioactive gas enrichment, such as iodine vapor, krypton (Kr), and Xenon (Xe), is also critical. Iodine species existing in solutions (such as IO_3_^−^ and I_3_^−^) and in the dissolver off-gas (DOG) stream are highly volatile diatomic elemental iodine (I_2_, 90%–100%) along with a small fraction of organic iodides (e.g., methyl iodide and ethyl iodide, 0%–10%) [19]. He et al. successfully synthesized N-rich COFs SCU-COF-2 and applied it to iodine gas removal [20]. SCU-COF-2 showed extremely high adsorption capacity for CH_3_I and I_2_ of 1.45 g/g and 0.98 g/g, respectively, and its iodine uptake outperforms commercial zeolites and active carbon under the practical dynamic scenario. Xe and Kr that are recoverable from the off-gases from used nuclear fuel (UNF) are of industrial importance. Zhang et al. synthesized robust MOFs (ECUT-60) with high chemical stability and multiple trap sites for Xe and achieved ultrahigh Xe adsorption capacity at room temperature [21]. When used for Kr/Xe separation, ECUT-60 showed unprecedented separation performance for tracing Xe/Kr in dynamic breakthrough experiments, leading to record Xe uptake up to 70.4 mmol/kg and the production of 19.7 mmol/kg pure Xe.

In summary, the following aspects should be considered in designing materials for efficient radionuclide removal including (ⅰ) extreme conditions of strong acid medium, high ionic strength, and strong ionizing radiation; (ⅱ) the effect of material properties on radionuclide removal (functional group, hydrophilicity, polarity, electrostatic interaction, and complexation); (ⅲ) the stability and recyclability of material; (ⅳ) the possibility of secondary contamination; (ⅴ) economic cost and practicability; and (ⅵ) material post-processing problem.

## 3 Emerging technologies for radionuclides removal

### 3.1 Adsorption technology

Adsorption technology, featuring low cost, simple operation, and strong practicality, has been extensively used in the removal of various radionuclides. The development and design of adsorbents with rich functional sites, large specific surface area, and strong stability is the key to adsorption technology (Fig. 1). Wang’s group from Soochow University has achieved remarkable progress in the design and application of MOFs, POPs, and polyoxometalate (POM) for radionuclides separation and removal [10,22]. For example, Sheng et al. reported a stable three-dimensional cationic MOF SCU-102, which showed fast adsorption kinetics, large capacity (291 mg/g), high distribution coefficient, and record-high uptake selectivity for ^99^TcO_4_^−^ removal [12]. The theoretical calculation revealed that the exceptional selectivity for ^99^TcO_4_^−^ benefited from the abundant hydrophobic pockets in the structure. Similar chemical properties make the separation of radionuclides difficult. Americium, as a radionuclide with long-term radiotoxicity, possesses similar ionic radii and coordination chemistry with lanthanides. Achieving the target of efficient removal and recovery of americium to reduce the harm to the environment is extremely challenging. Zhang et al. prepared a POM cluster with a vacancy site compatible with the selective coordination of hexavalent actinides (^238^U, ^237^Np, ^242^Pu, and ^243^Am) and discriminated against Ln(III) cations [23]. The obtained Am(VI)-POM clusters showed the strongest stability in aqueous solution so far. This pioneering research successfully achieved ultra-fast and efficient separation of lanthanum-hydrated and americium-hydrated ions, which made a major contribution to the sustainable development of nuclear energy and the management of hazardous radionuclides.

Besides adsorption technology, ion exchange, co-precipitation, etc., are also applied to the removal of radionuclides. However, a tough challenge is that these technologies easily induce the enrichment and separation of radionuclides without reducing their own harm, and require subsequent strict disposal.

### 3.2 Electrocatalysis technology

Compared with adsorption technology, electrocatalysis technology can achieve the target of continuous extraction of radionuclides through reduction or oxidation through electric fields (Fig. 1). Recently, Cui et al. reported an electrochemical deposition method to realize the continuous uranium extraction from spiked seawater [24]. By applying a square wave voltage of −5 V to 0 V, the adsorbed UO_2_^2+^ was firstly reduced to UO_2_ and subsequently oxidized to U(O_2_)O_2_·H_2_O solid in the air for facile collection. Based on this innovative research, our group successfully prepared Fe-N_x_-C-R and In-N_x_-C-R adsorption-electrocatalysts containing single-atom catalytic center and amidoxime (R) groups, both exhibiting distinguished uranyl affinities and extremely high uranium extraction capacity from seawater [25,26]. The FeN_x_ and InN_x_ catalytic center could rapidly electrocatalyze the adsorbed UO_2_^2+^ into Na_2_O(UO_3_·H_2_O)_x_ solid through a reversible U^6+^↔U^5+^ redox pathway, thereby realizing easy uranium product collection. We also reported adsorption-electrocatalysis Ru@HNCC-R loaded with ruthenium clusters and modified with a cationic polymeric network (R) containing imidazolium-N^+^ units, which achieved selective removal of ^99^TcO_4_^−^ (or its surrogate ReO_4_^−^) from extreme environmental conditions of high acidity, alkalinity, ionic strength, and radiation field [27].

Electrochemical technology is regarded as next-generation technology for sewage treatment because of its strong controllability, high efficiency, and environment friendliness. In particular, electrochemical technology can realize continuous reduction and fixation of radionuclides even in extreme conditions. The developed electrochemical technology offered uptake kinetics for radionuclides 2 to 3 orders of magnitude faster than traditional adsorption technology.

### 3.3 Photocatalysis technology

Besides electrochemical technology, the adsorption-photocatalysis system also exhibited prospects for selectively and efficiently radionuclides removal (Fig. 1). Photocatalytic technology can activate catalysts through light field resources, which has the advantage of eco-friendliness, mild reaction conditions, low cost, and efficiency, and has shown unique advantages in radionuclide removal in recent years. By introducing photo-active sites in the nanopores of COFs and amidoxime functional groups for uranyl binding, the extraction capacities for uranium up to 4.62 mg/g per day were achieved under visible light irradiation [28]. Moreover, the integration of donor–acceptor into COF structures is an effective strategy to enhance photocatalytic performance. For instance, a photocatalytic uranium extraction efficiency of 8.02 mg/g per day was realized using nitro-functionalized multicomponent COFs from natural seawater, exceeding the performance of all COF materials reported to date [11].

Photocatalyst technology reduces soluble radionuclides to insoluble products by photogenerated electrons, achieving efficient removal and separation of radionuclides. By adjusting the solution pH values and adding organics, U(VI) could form precipitates through disproportionation reactions under visible light irradiation and thereby be separated from solutions [29,30]. This method of integrating cooperative functions in photocatalysts demonstrates better efficiency compared to conventional materials for radionuclide removal.

### 3.4 Other technologies

Besides the methods mentioned above, membrane separation technology, with the advantages of simple operation, no chemical and phase change, strong adaptability and repeatability, and low energy consumption, also showed promising prospects in the separation of radionuclides [31]. Wang et al. prepared a novel graphene oxide (NPG) membrane with pillared-nanodiamond through a vacuum-assisted self-assembly method [32]. The NPG possessed increased interlayer space, narrowed channel-size distribution, enhanced thermal stability, and hydrophilicity. NPG membrane exhibited precise charge-discriminated group separation ability in a strong acidic condition containing ten coexisting cations. Specifically, the average filtration rates of the NPG membrane were improved by 76.8% for monovalent ions, 63.5% for divalent ions, 118.0% for trivalent lanthanide ions, 71.0% for UO_2_^2+^, and 105.1% for Th^4+^, respectively, compared with pristine GO membrane. The results of this study showed that the prepared membranes exhibited great application prospects in the separation of metal ions. In another study, Wang and co-workers realized the separation of actinides (U, Np, Pu, Am) and lanthanides (Ce, Nd, Eu, Gd, etc.) in nitric acid solution by using the prepared graphene oxide membrane [33]. Therefore, membrane separation technology showed broad application prospects in radionuclide separation.

## 4 Future perspective

Although substantial gains in the development of advanced materials and technologies for radionuclide removal have been achieved in recent years, an expanse of untapped potential remains in the materials design, technique exploration, mechanism study, and practical application for removing radionuclides. In order to fully explore this promising potential in the field of radionuclides management, some proposals should be made to address the challenges that currently hinder their practical application. (ⅰ) The existence forms of radionuclides are various and complicated in practical environments, including gases krypton (Kr), xenon (Xe), and I_2_ vapor, organic matter CH_3_I, positive ions Cs^+^, Sr^2+^, oxometallate ^99^TcO_4_^−^, UO_2_^2+^, etc., thereby the complexity of the environment should be fully considered when designing materials for radionuclide removal. (ⅱ) The cumbersome preparation process of advanced porous materials impedes their application in practical environments. Future studies should focus on developing affordable nanomaterials using environment-friendly and inexpensive raw materials, and improving synthesis methods to achieve large-scale preparation. (ⅲ) Incorporation of the merits of various technologies construct collaborative technology systems such as adsorption-electrocatalyst, adsorption-photocatalyst, and photo-electrocatalyst system to realize the objective of efficient, highly selective, continuous removal of radionuclides. (ⅳ) It is especially significant to gain insight into the structure of materials and the mechanisms of radionuclide capture at the molecular level, necessitating advanced characterization techniques such as *in situ* Raman, *in situ* FT-IR, and X-ray absorption spectroscopies (XANES/EXAFS). (ⅴ) There are many resource nuclides, such as U (raw material for nuclear energy), and I (medical resource), which should be properly recycled to promote the development of the nuclear energy cycle.

To summarize, exploring materials and technologies for radionuclides removal and extraction significantly promotes the development of nuclear energy, and is of great importance to human health and global ecosystems. To achieve this ambitious goal will demand the effective cooperation of environmentalists, chemists, and material scientists. We, therefore, call for comprehensive implementation of strategies and strengthened cooperation to mitigate the harm caused by radioactive contamination from FAN to oceans, atmosphere, soil, and human health.

## Author contributions

X.L.L.: investigation, writing–original draft. M.L.X., Y.L., Z.S.C.: investigation. H.Y.: writing–review & editing. X.K.W.: writing–review & editing, supervision. All authors contributed to the discussion, and gave approval to the final version of the manuscript.

## Declaration of competing interests

The authors declare no conflicts of interest.
